# An Initial Proteomic Analysis of Biogas-Related Metabolism of *Euryarchaeota* Consortia in Sediments from the Santiago River, México

**DOI:** 10.3390/microorganisms11071640

**Published:** 2023-06-23

**Authors:** Jesús Barrera-Rojas, Kelly Joel Gurubel-Tun, Emmanuel Ríos-Castro, María Cristina López-Méndez, Belkis Sulbarán-Rangel

**Affiliations:** 1 Department of Water and Energy, Campus Tonalá, University of Guadalajara, Tonalá 45425, Mexico; jesus.barrera@academicos.udg.mx (J.B.-R.); joel.gurubel@academicos.udg.mx (K.J.G.-T.); 2 Laboratorios Nacionales de Servicios Experimentales, Centro de Investigación y Estudios Avanzados del IPN, Ciudad de México 07000, Mexico; eriosc@cinvestav.mx; 3 Wetlands and Environmental Sustainability Laboratory, Division of Graduate Studies and Research, Tecnológico Nacional de México/ITS de Misantla, Veracruz 93850, Mexico; mclopezm@itsm.edu.mx

**Keywords:** archaea consortia, inoculum for biogas, proteomics, methane, archaeal metabolism

## Abstract

In this paper, sediments from the Santiago River were characterized to look for an alternative source of inoculum for biogas production. A proteomic analysis of methane-processing archaea present in these sediments was carried out. The *Euryarchaeota* superkingdom of archaea is responsible for methane production and methane assimilation in the environment. The Santiago River is a major river in México with great pollution and exceeded recovery capacity. Its sediments could contain nutrients and the anaerobic conditions for optimal growth of *Euryarchaeota* consortia. Batch bioreactor experiments were performed, and a proteomic analysis was conducted with current database information. The maximum biogas production was 266 NmL·L^−1^·g VS^−1^, with 33.34% of methane, and for proteomics, 3206 proteins were detected from 303 species of 69 genera. Most of them are metabolically versatile members of the genera *Methanosarcina* and *Methanosarcinales*, both with 934 and 260 proteins, respectively. These results showed a diverse euryarcheotic species with high potential to methane production. Although related proteins were found and could be feeding this metabolism through the methanol and acetyl-CoA pathways, the quality obtained from the biogas suggests that this metabolism is not the main one in carbon use, possibly the sum of several conditions including growth conditions and the pollution present in these sediments

## 1. Introduction

**Figure 1:**
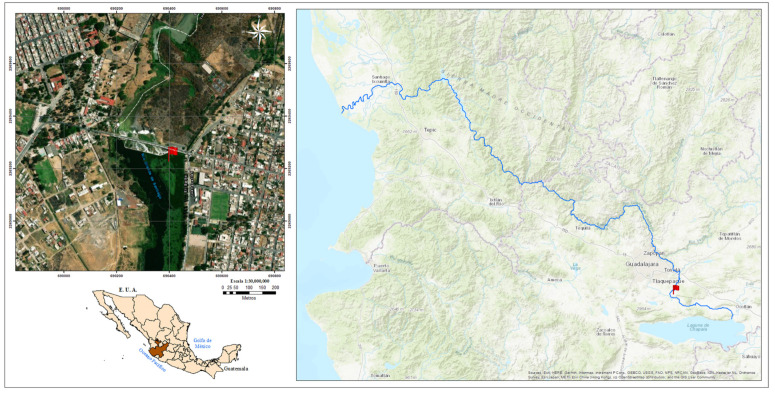
Localization of the Río Grande de Santiago in Jalisco, México. Top left, red flag indicates the sample site. Bottom left, localization of Jalisco state in México. Right, the course of the Río Grande de Santiago; red flag indicates the sample site.

The Río Grande de Santiago (Santiago River) is part of the Lerma–Chapala–Santiago Basin (LCS), and belongs to rivers with eastern slope, descending roughly 1700 m, from Jalisco State to the Pacific Ocean coast in Nayarit State (Figure 1). The Santiago River is eroding the Neo volcanic belt above the North America plate, flowing for 562 km. It rises between the Zula River and Chapala Lake (20°20′41.0″ N 102°46′44.1″ W), bordering the Metropolitan Area of Guadalajara (AMG; from its name in Spanish) across Santiago’s Canyon flowing from north to west over the Sierra Madre Occidental. The Santiago River is in Nayarit through La Yesca, feeds the hydroelectric power plant El Cajón, and finally discharges close to San Blas in the Pacific Ocean (21°38′20.9″ N 105°26′41.5″ W).

Chapala Lake is the central water body of LCS, can store 8126 billion liters of water, and its main tributary is the Lerma River. That and the Santiago River are two of the most polluted rivers in México [1,2]. Both rivers flow around the main urban areas in México: the Lerma River bordering the metropolitan area of the Mexican Valley (ZMVM) with 19.38 million habitants, and the Santiago River bordering the AMG with 5.26 million habitants [3]. These rivers receive not only municipal wastewater, but also industrial discharge, making treatment of water difficult and contributing to the increasing prevalence of diseases [4,5], social inequities [6], and visible environmental degradation. These anthropogenically imposed water conditions change the natural transfer of oxygen, stimulate the degradation of anaerobic organic matter, generate water toxic to most pluricellular organisms, and promote the proliferation of vectors such as the mosquito. This imbalance in water conducted across the Santiago River contributes to thriving bacterial and archaeal consortia. The sewer smell, disrupting organoleptic characteristics, the absence of fish and native birds, and mosquito swarms are the obvious indicators of environmental degradation in the Santiago River.

Not only is water quality affected by pollution in the Santiago River, but the organic matter load is also converted by anaerobic archaea into methane, a less persistent greenhouse gas, but more powerful than CO_2_. Methane is a reactive gas with chemical activity in the troposphere, mostly modifying hydroxyl concentrations [7]. Anthropogenic methane emissions rose 355,801 kilotons (kt) in 2022, agriculture being the primary source with 37% (141,954 kt), followed by energy (133,351 kt) and waste (70,759 kt) [8]. Besides that, with wetlands and oceans, sediments (lakes and rivers) produce 371,000 kt by natural processes [9].

Additionally to this, current archaeal genetic information has shown the diversity and global relevance of this root of the tree of life [10]. Research on archaea has gained interest because of the application of Clustered Regularly Interspaced Short Palindromic Repeats (CRISPR) technology in genetic engineering [11], the acquisition of thermostable enzymes [12], sustainable energetics because of their ability to produce methane [13], and other recent biotechnological applications [14]. In the environment, these microorganisms are relevant in the biogeochemical cycles of elements [15,16], such as carbon for methane production and consumption [17] as well as nitrogen [18,19]. In addition, the metabolic pathways of the archaea domain are diverse in the use of carbon sources [20,21], processing of membranes [22,23], nucleic acids [24,25], protein activity, processing [26,27], electron transport [20,28,29], and, recently, the identification of a putative nucleolus structure in the crenarchaeon *Saccharolobus solfataricus* related to ribosome biogenesis [30]. Given the importance of study of the archaea, the present research aimed to characterize biogas production and determine the proteins present in the sediments of the Santiago River related to methane-processing metabolisms. The sediments of the Santiago River could be used as alternative inoculums in an anaerobic digestion system. For this, experiments were carried out to evaluate the volumetric yield of biogas per gram of microorganisms as volatile solids from the ratio of methane in biogas. Using a handmade euryarcheotic protein database and mass spectrometry, it was possible to determine the main species and metabolisms present in the sediment of the Santiago River; using this information, the pathways of methane production will be discussed.

## 2. Materials and Methods

### 2.1. Sediment Sampling

A sampling site (20°30′45.1″ N 103°10′26.4″ W) was selected near “El Salto de Juanacatlan”, Jalisco (Figure 1). To obtain anaerobic sediment, a backhoe loader was used. It excavated 3 m deep to obtain three samples. Samples were stored on ice, and 45 mg/L of NaHSO_3_ was added to each. Following that, the sediment samples were transported to the laboratory, and stored at 4 °C for further analysis.

### 2.2. Physicochemical Characterization of Sediments

The samples of sediments from the Santiago River (SRS) were characterized by measuring the following parameters in situ using a YSI 1725 water quality meter: pH, oxidation–reduction potential (ORP), electric conductivity (EC), salinity, and total dissolved solids (TDS). Chemical oxygen demand (COD), total organic carbon (TOC), total solids (TS), and volatile solids (VS) were determined in laboratory facilities using colorimetric Hach probes for the first two (2125915 LM, 2815945, respectively) in DR900, and the gravimetric technique for the last two. Dissolved oxygen was measured using an electrode.

For heavy metal determination, atomic absorption spectrophotometry equipment PerkinElmer AAnalyst 200 was used. The sediment sample was ground and preserved by adding nitric acid until it reached a pH of less than 2 and kept at 4 °C until analysis. Three sample replicates were filtered, and residues were vacuum-dried and sieved to a particle size of 2 mm. A mixture of HCl:HNO_3_ at a 1:3 ratio was used for digestion using an autoclave. Concentrations were interpolated using high-purity NIST-traceable standards. For the determination of arsenic, a hydride generator included in the PerkinElmer AAnalyst 200 flame atomic absorption spectrophotometry equipment was used.

### 2.3. Batch Bioreactor Experiments

The samples of SRS were diluted in a culture medium to a final concentration of 20 g/L VS. The culture medium was formulated according to previous independent work [31], with the following modification: 1.5 g/L NaCl, 18.69 mM NH_4_Cl, 0.8 g/L MgSO_4_·7H_2_O, 0.6 g/L MnSO_4_·H_2_O, 0.5 g/L FeSO_4_·H_2_O, 0.5 g/L KH_2_PO_4_, 0.5 g/L K_2_HPO_4_, 50 mg/L KCl, 50 mg/L CaCl_2_·7H_2_O, 20 mg/L CuSO_4_·5H_2_O, 0.5 mg/L Na_2_MoO_4_·2H_2_O, 1.5 mg/L NiCl_2_·6H_2_O, 0.1 mg/L ZnSO_4_·7H_2_O, 7 µg/L H_3_BO_3_, 4 µg/L CoSO_4_·7H_2_O, 4 µg/L Na_2_WO_4_·2H_2_O, 3 µg/L Na_2_SeO_3_·5H_2_O, and 0.2 µg/L KAl(SO_4_)_2_·12H_2_O. pH was adjusted to 7, and after autoclaving the medium, the following filtered compounds were added: 2 g/L NaHCO_3_, 27.7 mM butyric acid, 68 mM methanol, and 25.5 mM CH_3_COONa. The bioreactor was bubbled with N_2_ (CAS: 7727-37-9) for 10 min to displace oxygen. Experiments were carried out in a bath wash at 37 °C under constant stirring and in an instrumented bioreactor Minifors 2 with pH, temperature, and agitation control.

### 2.4. Methane Determination

Biogas was passed through a 2 M NaOH solution and stored in gasbags. Biogas flow was measured with a µFlow Gas Meter (Bioprocess Control, Lund, Sweden) with real-time pressure and temperature compensation. Methane composition was determined with Clarus^®^ 580 gas chromatography equipment (PerkinElmer, Waltham, MA, USA) equipped with a HayeSep D column (3 m × 3.2 mm; 100/120 mesh) connected to a thermal conductivity detector operated at 120 °C. Biogas samples (500 μL) were injected (injector temperature 75 °C) and carried with an N_2_ flow of 30 mL/min. Curves with different amounts of pure methane were used as references to interpolate the methane concentration in biogas samples.

### 2.5. Whole Cell Extract

All the following procedures were carried out at 4 °C. Once the biogas production reached the stationary phase after 213 h, aliquots corresponding to 2 g of VS from SRS were subjected to centrifugation in 0.25 M of sucrose solubilized in buffer A (50 mM Bis-Tris pH 7.5, 100 mM NaCl, 1% sodium azide, 1 mM of PMSF) at 16,000 rpm for 15 min. Pellets were resuspended in buffer A with 20 mM dithiothreitol (DTT) and 1% sodium dodecyl sulfate (SDS). Resuspended cells (50 mL) were disrupted using an Ultrasonic Processor (Cole-Palmer). Intervals used were 5 s (on) and 30 s (off) for 48 cycles at 50% amplitude. Lysate was centrifugated at 12,000 rpm for 20 min. Part of the supernatant was incubated with 10% trichloroacetic acid for 20 min. On the other hand, the supernatant was mixed with 4 volumes of cold acetone (−20 °C). Both samples were centrifugated at 16,000 rpm for 20 min. Precipitated proteins were resuspended with an aliquot of buffer A with 7 M urea and 0.1% SDS and stored at 4 °C for further analysis.

### 2.6. SDS-PAGE of Whole Cell Extract

A 50 μg aliquot of proteins from whole cell extract was mixed in equal proportion with digestion buffer (100 mM Tris base pH 8, 2.5% (*w*/*v*) SDS, 20 mM DTT, 30% (*v*/*v*) glycerol, and 0.02% (*w*/*v*) bromophenol blue) and incubated for 20 min at 60 °C in a water bath, cooled down at room temperature, and loaded onto 12% polyacrylamide gel to carry out electrophoretic migration of proteins. The gel was stained with Coomassie Blue R-250. Molecular weight markers corresponding to purified bovine serum albumin (BSA; 65 kDa), ovalbumin (45 kDa), chymotrypsin (25 kDa), egg lysozyme (14.3 kDa), and horse cytochrome C (12.5 kDa) were used.

### 2.7. Mass Spectrometry-Based Proteomics

A 40 µg aliquot of protein was loaded in a 12% 1D SDS-PAGE gel, separated ≈1 cm inside the resolving gel to concentrate the proteins in a small gel zone, and dyed with Coomassie Blue. After the electrophoretic run, gels were digested “in-gel” [32] with some modifications. Briefly, gel slides were excised from the gel and cut into ≈1 mm pieces; cut bands were transferred into centrifuge microtubes to be destained with 2.5% formic acid (FA) and 50% MeOH, they were subsequently dehydrated with acetonitrile (ACN), and the remaining solvent was eliminated in a Savant DNA120 SpeedVac Concentrator (Thermo Fisher Scientific, Waltham, MA, USA) for 10 min. Then, proteins contained in dried bands were reduced with 10 mM DTT (Sigma-Aldrich, St. Louis, MO, USA) in 100 mM ammonium bicarbonate (ABC) (Sigma-Aldrich) and alkylated with 50 mM iodoacetamide (IAA) (Sigma-Aldrich) in 100 mM ABC. Afterward, cut bands were washed with 100 mM ABC and dehydrated with ACN; subsequently, cut bands were hydrated and washed anew with 100 mM ABC and dehydrated with ACN; then, the excess solvent was removed using the SpeedVac for 10 min. Proteins in gel were enzymatically digested overnight using 20 ng/µL trypsin (Sigma-Aldrich) in 50 mM ABC at 37 °C in a Precision water bath (Thermo Fisher Scientific, Waltham, MA, USA). Once the time had passed, the reaction was stopped with 40 µL of 5% FA for 10 min at room temperature; subsequently, peptides were eluted from the gel for two cycles using 40 µL of a solution of 5% FA and 50% ACN. Peptides were concentrated in the SpeedVac and desalted using Pierce^TM^ C18 spin columns (Thermo Fisher Scientific, Waltham, MA, USA); finally, the resulting peptides were again concentrated and an aliquot from a stock of BSA (UNIPROT accession P02769; Waters, Milford, MA), was added, as an internal standard, to tryptic peptides to obtain a final concentration of 25 fmol/μL (final volume of 40 µL) and stored at −20 °C until liquid chromatography-coupled mass spectrometry (LC-MS) analysis.

The LC-MS analytical method was applied according to Ríos-Castro et al. with some modifications [33]. Briefly, 3.5 µL of tryptic peptides was loaded and separated on an HSS T3 C18 column (Waters, Milford, MA, USA; 75 μm × 150 mm, 100 Å pore size, 1.8 μm particle size) using an ACQUITY M-Class UPLC with the following mobile phases: A, 0.1% FA in water; and B, 0.1% FA in ACN. The following gradient was applied for mobile phases: 0 min 7% B, 121.49 min 40% B, 123.15 to 126.46 min 85% B, and 129 to 130 min 7% B. The gradient flow was set at 400 nL/min and 45 °C. The spectral data were acquired in a Synapt G2-S*i* mass spectrometer (Waters, Milford, MA, USA) using nano-electrospray ionization (nano ESI) and ion-mobility separation (IM-MS) using a data-independent acquisition (DIA) approach through a high-definition multiplexed MS/MS (HDMSE) mode. For the ionization source, parameters were set with the following values: 2.75 kV in the sampling capillary, 30 V in the sampling cone, 30 V in the source offset, 70 °C for the source temperature, 0.5 bar for the nanoflow gas, and 150 L/h for the purge gas flow. Two chromatograms were acquired (low- and high-energy chromatograms) in the positive mode in a range of *m/z* 50−2000 with a scan time of 500 ms. No collision energy was applied to obtain the low-energy chromatograms while for the high-energy chromatograms, the precursor ions were fragmented in the transfer cell using a collision energy ramp from 19 to 55 eV.

### 2.8. MS Analysis

The MS and MS/MS measurements contained in the generated *.raw files were analyzed and absolutely quantified by Progenesis QI for Proteomics software v4.2 (Waters, Milford, MA, USA) [34] using a target-decoy strategy against an archaea database containing the following orders and number of sequences (in brackets) downloaded from UniProt: *Methanomassiliicoccales* (69,548), *Methanocellales* (13,887), *Methanosarcinales* (404,689), *Methanomicrobiales* (215,651), *Methanobacteriales* (233,508), *Methanocellales* (70,148), *Methanopyrales* (3228), *Archaeglobales* (66,748), *Thermoplasmatales* (96,439), and *Halobacteriales* (552,907). Sequences were concatenated in reverse sense in the same *.fasta file to apply the target-decoy strategy to deliver false-positive estimations [35,36]. Parameters used for the protein identification were trypsin as cutting enzyme and one missed cleavage allowed; carbamidomethyl (C) as a fixed modification and oxidation (M), amidation (C-terminal), deamidation (Q, N), and phosphorylation (S, T, Y) as variable modifications; default peptide and fragment tolerance (maximum normal distribution of 10 and 20 ppm, respectively), and false discovery rate ≤ 1%. All false-positive identifications (reversed proteins) and proteins with one peptide identified were discarded for subsequent analysis. Synapt G2-S*i* was calibrated with [Glu1]-fibrinopeptide fragments, through the precursor ion [M + 2H]^2+^ = 785.84261 fragmentation of 32 eV at 1 ppm across all MS/MS measurements. Quantification of proteins was calculated based on the average MS signal response of the three most intense tryptic peptides (*Top*3) [37], according to Equation (1) [37,38].

(1) $$Top3=\frac{i1+i2+i3}{3}$$

where *i*1, *i*2, and *i*3 are the spectrometric counts of the most intense peptides.

(2) $$USRF=\frac{Top3}{f{mol}_{BSA}}$$

With Equation (2), the universal signal response factor (*USRF*) was determined. *fmol*_BSA_ is the amount (femtomoles) of BSA injected into the LC column. Then, with Equation (3), *fmol* for each of the quantified proteins was determined.

(3) $$fmol=\frac{{Top3}_{x}}{USFR}$$

where *Top*3*_x_* is equal to the top three values of each identified protein in the sample.

### 2.9. Protein Data Analysis

Graphs were generated with R code; cellular components, biological processes, and molecular processes were obtained using the UniprotR package [39] from the UniProt database.

## 3. Results

### 3.1. Volumetric Methane Yield by Archaeal Consortia in SRS

The use of manure to inoculate bioreactors is a common practice; it corresponds to the presence of anaerobic consortia in cattle guts to ensure methane production [40,41]. Looking for an alternative source of inoculum, SRS were used. Parameters of SRS measured in situ showed appropriate conditions to carry out methanogenesis: neutral pH, low redox potential, and a low dissolved oxygen concentration (Table 1). Subsequent laboratory characterization showed an organic-rich sediment with 58.88 g VS/L, and a total COD of 34,200 mg/L, but a relatively low soluble COD of 237 mg/L. The C/N ratio was close to 1, using TOC and total nitrogen measured in filtered water. VS suggest an abundance of aggregated biomass, but low dissolved organic carbon is available as a carbon source in the aqueous phase of SRS.

Interestingly, in the analysis of metals in the sediment, we found a high concentration of arsenic, suggesting that in the proteomic analysis we might find proteins related to arsenic metabolism; however, we did not obtain any results within our criteria of reliability. This suggests that arsenic is found in the mineral fraction of the sediment and there is no significant release that promotes the expression of genes related to arsenic processing.

**Figure 2:**
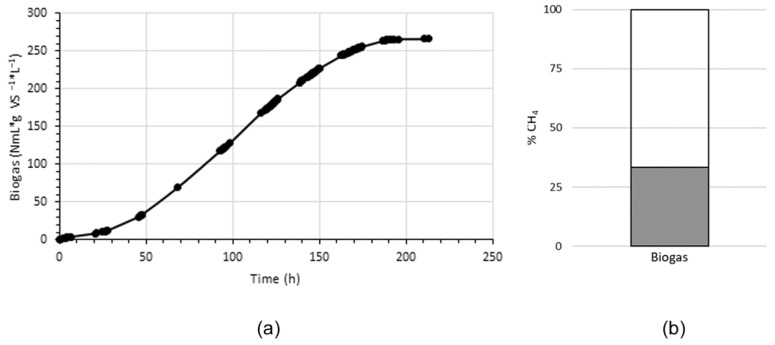
(**a**) Specific biogas yield and (**b**) methane concentration. NmL, normal biogas volume adjusted at temperature and pressure values; g VS corresponds to biomass in the bioreactor including microorganisms and non-identifiable biomass detritus at the beginning of the experiment.

Figure 2 shows a batch experiment for biogas production. The maximum biogas production was 266 NmL*L^−1^*g VS^−1^ with 33.34% of methane. The volume of biogas produced was in accordance with that of other reports on the use of sediments as inoculum [42,43]; however, in the experiments, the stationary phase was reached in a relatively short time. The low quality of biogas determined by the methane content obtained in the first batch experiments could be attributed to several factors that will be discussed.

### 3.2. Best-Represented Proteins

Immediately after that methane production experiment was finished, a sample was taken, and proteomic analysis was carried out. Disrupting the cellular structure of archaea requires chaotropic ions, solubilizer molecules, reducing agents, and mechanical energy. Once proteins are obtained by precipitation with trichloroacetic acid (TCA) and cold acetone (Figure S1), proteolytic digestion with trypsin permits a reduction of the information of protein identities into small blocks of peptides starting or ending with the constant amino acid residues lysine and arginine. Fragmentation of these peptides was carried out with non-collision and collision energy ramps for identification of amino acid residues by sophisticated data acquisition; mass spectrometry showed 3206 proteins with more than two peptides detected (P) and more than one unique peptide (UP). The best-represented proteins are those with a greater number of peptides detected; below we describe the top four proteins and their complexes in SRS samples.

**Figure 3:**
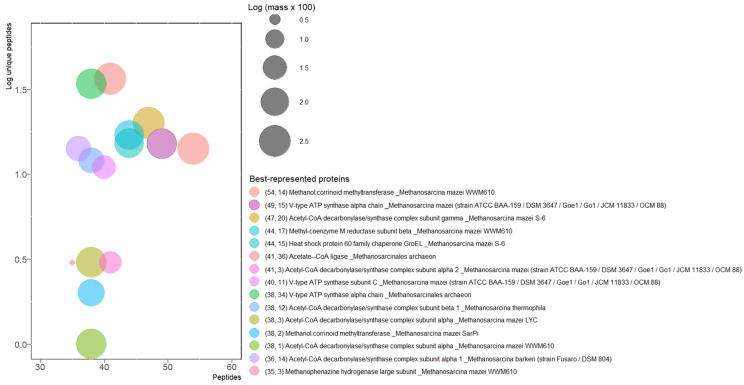
Best-represented proteins. To prevent negative values, mass was multiplied by 100, and log10 was applied; corresponding values are in Table S2. In brackets, total peptides and unique peptides, respectively.

The best-represented protein in SRS was methanol:corrinoid methyltransferase (MtaB, UniProt ID A0A0E3LEL4; 54 P, 14 UP) of *Methanosarcina mazei* WWM610 (Figure 3). It is part of the complex that catalyzes the disruption of methanol (MtaA–MtaB) and the transfer of a methyl group to the cob(I)alamin cofactor of MtaC. Finally, MtaC transfers the methyl to coenzyme M. We obtained 4, 10, and 3 protein identifications of MtaA, B, and C, respectively, from different species (EC 2.1.1.90 in KEGG). The presence of MtaB corresponds to the use of methanol in our culture medium (68 mM) by *M. mazei* WWM610, and its abundance (36th place of most abundant individual proteins by mass, Table S1) suggests that in the starving conditions of the SRS, the most digestible carbon source is monomethylated one-carbon alcohol [44].

The second best-represented protein was the α (or A) subunit of the V-type ATP synthase (V-ATPase) of *M. mazei* (A0A0F8P8C6; 49 P, 15 UP). These complexes are molecular rotatory motors that use the electrochemical potential (protons or sodium) across the membrane to produce ATP. Usually, the V-type (from vacuolar) corresponds to complexes in eukaryotic cells acidifying vacuoles by ATP hydrolysis. However, the closely related sequences and distribution across bacteria, eukarya, and archaea domains go beyond mere names [45]. V-types in archaea contain α (A), β (B), C, D, E, F, I, K, and G/H subunits (M00159 module in KEGG); in SRS, we detected the first eight. In total, 86 proteins were identified, almost all annotated as V-ATPase, only 1 as ATP synthase, and 2 as F0F1 ATP synthase (Table S3). In SRS, the V-ATPase is the fourth most abundant by mass of protein (all ortholog subunits detected, Table S4).

Following on to the CO_2_ pathway, the third best-represented protein was the γ subunit of acetyl-CoA decarbonylase/synthase (CODH/ACS) of *M. mazei* S-6 (A0A0E3LTN3; 47 P, 20 UP). In addition, five subunits of CODH/ACS from some *Methanosarcina* species were detected: the α2 subunit (A0A0E3LTN4; 41 P, 3 UP) of *M. mazei* Goe1, β1 subunit (A0A0E3NLE4; 38 P, 12 UP) of *M. thermophila*, δ subunit (A0A0E3LEU3; 26 P, 5 UP) of *M. mazei* WWM610, and ε subunit (A0A0E3LSW0; 19 P, 8 UP) of *M. mazei* SarPi (Table S5). This complex has been reported in the oligomeric form α_6_β_6_γ_6_δ_6_ε_6_ and catalyzes the formation/cleavage of the acetyl C–C bond of acetyl-CoA by an Ni and Fe_4_S_4_ catalytic site using a two-electron reduction with a negative potential of −495 mV [46].

As expected for an anaerobic microbial consortium, the central enzyme in methane production, methyl coenzyme M reductase (Mcr), was the fourth best-represented protein. The subunit protein corresponds to the β subunit (A0A0E3PU26; 44 P, 17 UP) of *M. mazei* WWM610. Mcr catalyzes the final reductive step in methane production by methanogenic archaea and the first oxidative step in methane degradation by archaeal methanotrophs (other results will be presented later).

Translation of mRNA into protein produces a linear sequence of amino acids, but in the end, the protein function depends on its three-dimensional folding. There are many multi-domain proteins, even in prokaryotic organisms with relatively small genomes that require fine-tuning mechanisms to prevent aggregation and obtain functional proteins [47]. Heat shock protein 60 (HSP 60) was the next best-represented protein (A0A0E3LTE9; 44 P, 15 UP) in SRS. It belongs to a family of constitutively expressed proteins that functionally are chaperones of protein maturation, HSP 60 being a catalyst of protein folding [48]. HSP 60 (58.32 kDa) forms a two-stacked ring, each with 7 proteins [49], with 14 in total. To carry out its functions, the structure of HSP 60 contains an unfolded protein-binding domain and an ATP-binding domain.

### 3.3. The Final Step of Methane Production: Methyl Coenzyme M Reductase

Bioenergetics pathways have crucial enzymes that play a main role by their abundance, catalytic activity, structure, and location, among other characteristics: ribulose-1,5 bisphosphate carboxylase-oxygenase (RuBisCO), as well as photosystems I and II, in photosynthesis; NADH dehydrogenase (complex I) and the cytochrome c oxidase (complex IV) in oxidative phosphorylation; nitrogen synthetase in nitrogen fixation; and the ubiquitous ATP synthase that releases ion-motive gradients across membranes producing ATP. Mcr is the enzymatic complex with the same status in methanogenesis and oxidation of methane (AOM). Mcr is an ellipsoidal-shaped hexamer in an α_2_β_2_γ_2_ arrangement with a molecular weight of approximately 300 kDa. It contains two active sites with coenzyme F430, a porphyrin-like structure coordinating a nickel atom. The catalytic site has five amino acid residues with post-translational modifications [50]. Mcr carries out the reversible oxidation (reduction) of methyl coenzyme M (CH_3_-S-CoM) and coenzyme B (HS-CoM) to release CH_4_ and the disulfide-linked molecule CoM-S-S-CoB. In SRS, 57 subunits of Mcr were detected from 28 species (Table S6). Besides the β subunit (A0A0E3PU26), other well-represented proteins belonging to the genus *Methanosarcina* are the α subunit (A0A0E3RG71; P 30, UP 2) of *M. mazei* S-6 and β subunit (A0A0E3LED7; P 30; UP 11) of *M. siciliae* C2J. Interestingly, three proteins of Mcr from methanotrophs were detected: subunit α (A0A7H1KNU1; P 2, UP 2) and the A2 component (A0A419JG48; P 4, UP 1) of the archaeal cluster ANME-2, and subunit γ (A0A284VQI0; P 4, UP 1) of Ca. *Methanoperedens nitroreducens*.

### 3.4. Most Abundant Proteins

**Figure 4:**
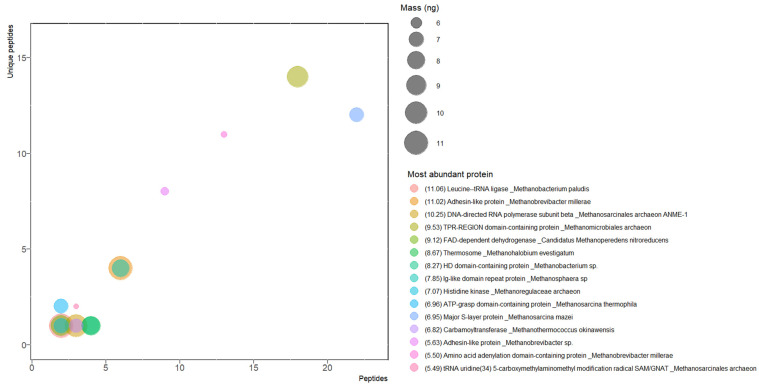
Most abundant proteins. Masses are symbolized by sphere size, and the values are shown in brackets. The values are in ng.

**Figure 5:**
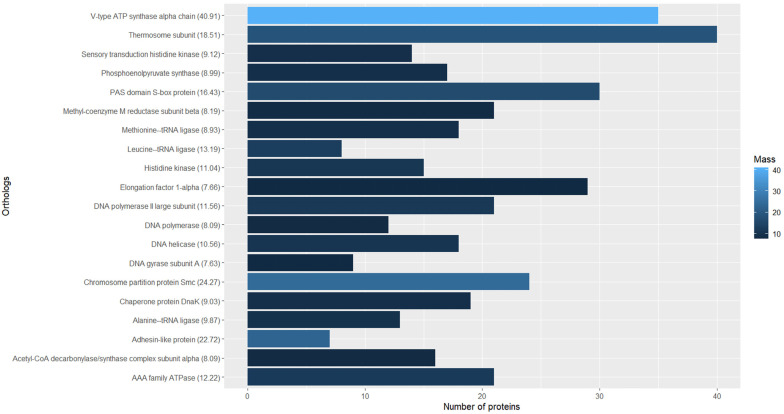
Most abundant orthologs in SRS. Masses (ng) are indicated in the color gradient and in brackets.

The current accuracy and sensitivity of the mass spectrometer allowed us to identify peptides in the femtomolar range, which means that nanograms are enough to get a confident identification of a considerable number of proteins in complex samples. Matching the femtomoles of peptides with the molecular mass of protein, the abundance was analyzed by proteins (Figure 4) and by orthologs in SRS (Figure 5).

The most abundant protein was leucine-tRNA ligase (F6D7Q0, 11.06 ng) of *Methanobacterium paludism* SWAN-1. This hydrogenotrophic methanogen has been isolated from two different peatlands, in New York and Alaska [51]. This housekeeping enzyme catalyzes binding (aminoacylation) of transfer ribonucleic acid (tRNA) with its cognate amino acid leucine, hydrolyzing ATP in the process.

With a very similar amount, the second most abundant protein was adhesin-like protein (A0A0U3E3W2, 11.02 ng). This membrane protein (407.5 kDa) was predicted from the genome of *Methanobrevibacter millerae* as a probably hydrogenotrophic methanogen detected in a rumen sample [52]. As for other species of this genus, it contains numerous adhesin-like proteins (up to 5% of its genome), which could have relevance in symbiotic associations with protozoa and bacteria [53].

On the other hand, the orthologs were grouped, and it was found that the V-type ATP synthase alpha chain was the most abundant protein with 40.91 ng from 35 species (Figure 5). Coupled with the β subunit it forms the V_1_ domain of ATP synthase in an oligomeric arrangement of (αβ)_3_. In the V_1_ domain, ADP and Pi form ATP by a conformational movement promoted by the V_0_ domain when the electrochemical potential is released in the membrane.

The second most abundant protein was the chromosome partition protein structural maintenance of chromosomes (Smc) (Figure 5). Working with other associated proteins, its function is related to ensuring the integrity of chromosome structure, replication, and conformation [54]. These ATPase protein complexes are found in bacteria and eukarya too; in SRS, 24 species adding to 24.54 ng were detected. Interestingly, the third group of orthologs is the adhesin-like protein family with only seven proteins from five species, all of them from *Methanobrevibacter*, with the amount totaling 22.72 ng. In addition, 267 uncharacterized proteins were detected in SRS (Table S4).

### 3.5. Main Coenzymes in Methanogenesis: CoB and CoM (Tetrahydromethanopterin)

Coenzyme B (7-mercaptoheptanoylthreonine phosphate, CoB) is a redox-potential carrier that donates two electrons to reduce the methyl group in CoM, forming a disulfide bridge CoM–CoB, and releasing methane as a byproduct of catalysis by Mcr. Besides that, anaerobic methanotrophic archaea (ANME) reduce CoB–CoM using a reversible methane-producing pathway to obtain CO_2_ and use a versatile electron-transferring pathway to grow in a methane-rich environment [29]. CoB biosynthesis starts with the use of 2-oxo adipate by homocitrate synthase to produce (R)-(homo)2-citrate. This step is followed by dehydration to obtain cis-(homo)2-aconitate. The presence of four proteins related to this metabolism was found in SRS, annotated as *aks*A gene product, of *Methanosphaera* (sp. WGK6 and sp. A6), *Methanoculleus* sp. SDB, and *Methanothermococcus okinawensis*, all of them with an average molecular mass of 41.84 kDa (A0A1D2X3K9, A0A1D2WC96, A0A0Q0VCJ9, and A0A832YSN9, respectively; Table S7). There are four intermediary molecules in the biosynthetic pathway of CoB whose enzymes are so far unknown: 2-oxooctanedionic acid, 7-oxoheptanoic acid, 7-mercaptoheptanoic acid, and 7-mercaptoheptanoylthreonine. The last one is the substrate for CoB biosynthesis by ATP:7-mercaptoheptanoylthreonine 3-phosphotransferase, which was not detected in SRS (M00608 module in KEGG).

Coenzyme M (2-mercaptoethanesulfonic acid, CoM) is a lipophilic coenzyme related to methane-forming and methane-consuming metabolisms in *Euryarchaeota*. CoM is the smallest coenzyme known (156.2 Da), which concentrates and carries methyl groups from several pathways to feed methane production. The biosynthetic pathway of CoM is not complete yet [55], but some enzymes have been described in detail. In the SRS, we only found the first enzyme related to CoM biosynthesis, the phosphosulfolactate synthase (ComA) of *Methanobrevibacter curvatus* (A0A166DZP7; 2 PD, 1 UPD; Table S7). ComA has activity removing sulfite from (2R)-O-phospho-3-sulfolactate to synthesize phosphoenolpyruvate (PEP). This enolase that is not an enolase [56], and uses Mg^2+^ to coordinate an enolate intermediate as the enolase superfamily, but its sequence is different, having a TIM-barrel fold (PDB 1QWG). Mg^2+^ ions and substrate, like ComA, are used by the following enzyme involved in CoM biosynthesis, 2-phosphosulfolactate phosphate (ComB), which produces (2R)-3-sulfolactate and ortho-phosphate. Interestingly, ComB ortholog genes are conserved among bacteria and cyanobacteria [57]. The product from ComB is oxidated by L-2-hydroxycarboxylate dehydrogenase (ComC) and reducing NAD^+^. It has been reported that ComC can act on multiple hydroxy carboxylates across archaeal metabolism [58]. The following enzyme is sulfopyruvate decarboxylase (ComDE) which produces sulfoacetaldehyde (SAA), releasing carbon dioxide. This thiamine diphosphate-dependent enzyme is an α6β6 dodecamer, which has been shown to be very sensitive to oxygen [59]. Although it seems that there are five intermediary steps in CoM biosynthesis from PEP, only the first four of them have been described; still missing is the change of aldehyde in SAA to a sulfhydryl group in CoM (M00358 module in KEGG).

### 3.6. Euryarchaeal Orders in SRS

**Figure 6:**
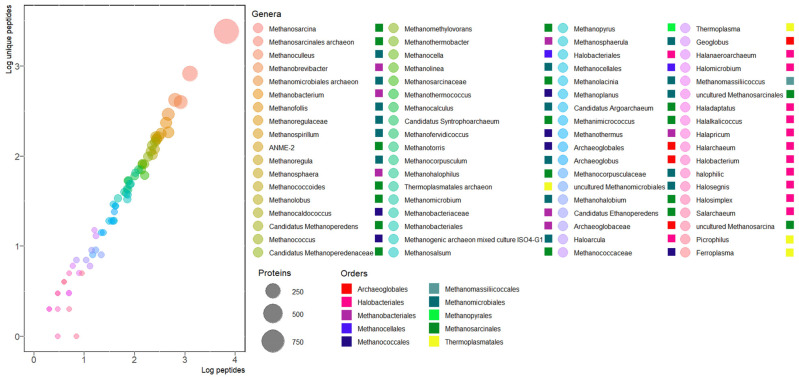
Euryarchaeal orders in SRS. Colored circles indicate the genera and squares the order.

In SRS, the three main orders detected were *Methanosarcinales* with 1657 proteins, followed by *Methanomicrobiales* with 779, and *Methanobacteriales* with 434 (Figure 6). The order *Methanosarcinales* is composed of versatile species with the ability to produce methane using diverse carbon sources and metabolisms such as: CO_2_-reducing hydrogenotrophic, methyl-dismutating, acetoclastic, methyl-reducing hydrogenotrophic, alkanotrophic, methoxyl-dismutating, and methanotrophic sources [20]. In SRS, 199 proteins of *Methanosarcinales archaeon* were detected. The *M. archaeon* genome was assembled from mangrove sediment samples, where the hydrogenotrophic *Methanomicrobiales* were the most abundant and the methylotrophic *Methanomassiliicoccales* was the most active order [60]. In SRS, only two proteins of *Methanomassiliicoccales* were detected. *Methanosarcina mazei* WWM610, another species of the order *Methanosarcinales*, was detected with 175 proteins, including the best-represented MtaB (A0A0E3LEL4). This archaeon was isolated from river sediments, and its genome was sequenced and used to identify the genomic and phenotypic diversity in the species *M. mazei* based on using trimethylamine as a carbon source [61].

*Methanomicrobiales* is a diverse order with the ability to reduce CO_2_ with H_2_ but cannot use methylated C-1 molecules as methanol, as well as acetate. For this order, in SRS, *Methanomicrobiales archaeon* was the species with the most proteins detected, with 80 being found. The *M. archaeon* genome was sequenced in a metagenomic study from groundwater samples to predict the impact of deep-subsurface sediment consortia in radioactive waste repositories [62]. In SRS, the best-represented (18 P, 14 UP) and most abundant protein (19.22 ng) from *M. archaeon* was a hypothetical TPR domain-containing protein (A0A2I0PCX3) with a molecular mass of 516.17 kDa. The TPR family (tetratricopeptide of 34-amino acid repeated motif) is associated with RNA processing and cellular division [63].

The order *Methanobacteriales* has a limited range of carbon sources and generally reduces CO_2_ with H_2_; some of its species can use secondary alcohols, formate, and CO. In SRS, the most abundant proteins belong to *Methanobrevibacter* sp., with 175 identifications. This organism has been found as part of the gastrointestinal tract microbiome of ruminants, being more abundant in the small intestine, playing a role in functional homeostasis and anaerobic digestion [64].

### 3.7. Euryarchaeal Methanotrophs

**Figure 7:**
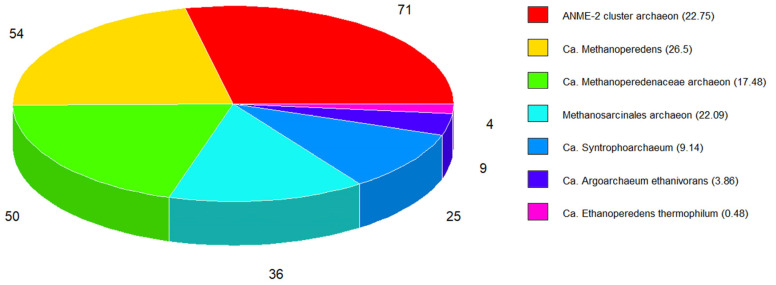
Genera of anaerobic methanotrophic archaea detected in SRS. In brackets, the mass of all the proteins detected in SRS. Numbers in the pie chart correspond to the number of proteins.

In SRS, 17 species of methane-oxidizing archaea were detected, with 249 proteins (Figure 7 and Table S8). For ANME-2 (cluster), the most abundant protein was an Smc protein (A0A3R7DNC8, 5.23 ng), and for ANME-2 HR1, the most abundant was DNA helicase (A0A2P5K5C7, 1.08 ng).

For Ca. *Methanoperedens*, three species were detected: Ca. *M. nitroreducens* with the oxidoreductase FAD-dependent dehydrogenase (A0A062VE78, 9.12 ng), Ca. *Methanoperedens* sp. with the ATP-dependent chaperone ClpB (A0A6A2FHA4, 0.84 ng), and Ca. *Methanoperedens* sp. BLZ1 with an Smc protein (A0A0P8CIV0, 3.18 ng).

For Ca. *Methanoperedenaceae*, four candidate species were identified. The most abundant of all was the aminoacyl-tRNA ligase Glu-tRNA amidotransferase (GatE, A0A842Y2C6, 1.42 ng) of Ca. *M. archaeon*. For Ca. *M. archaeon* GB37, the most abundant was the ATP-dependent serine protease Lon protease (A0A7R9NFT0, 0.61 ng). For Ca. *M. archaeon* GB50, the most abundant was the serine/threonine-protein kinase PknD (A0A7R9NAB8, 1.27 ng). Finally, for Ca. *M. archaeon* HGW, the most abundant was the subunit β of the acetyl-CoA decarbonylase/synthase complex (A0A2I0NJZ7, 0.4 ng).

For *Methanosarcinales archaeon*, ANME-1 ERB6, ANME-1 ERB7, and ANME-2c ERB4, 36 proteins were detected. The most abundant proteins were an uncharacterized protein of 123 kDa (A0A7G9YVX1, 1.43 ng), a DNA-directed RNA polymerase (β subunit, A0A7G9Z668, 10.25 ng), and a hypothetical protein with unknown function, DUF1156 domain-containing protein (A0A7G9YGH4, 0.76 ng), respectively.

The following close relatives of methanotrophs were detected in SRS. For the alkanotrophic Ca. *Syntrophoarchaeum*, three species were detected: Ca. *S. butanivorans* with a helicase activity DUF3883 domain-containing protein (A0A1F2P3G2, 2.99 ng), Ca. *S. caldarius* with a K^+^-insensitive pyrophosphate-energized proton pump (A0A1F2PC40, 0.62 ng), and Ca. *Syntrophoarchaeum* sp. GoM_oil with a DNA-binding protein— the EcoKI restriction-modification system protein HsdS (A0A564QAC7, 0.35 ng). Interestingly, these species use reversible methanogenesis enzymes to use butane as a carbon source [65]. On the other hand, nine proteins of Ca. *Argoarchaeum* were detected, the most abundant being phosphatase 2C (A0A811TCJ5, 1.21 ng), and four proteins of Ca. *Ethanoperedens* were detected, the most abundant being AAA family ATPase (A0A848D8W8, 0.15 ng).

### 3.8. Biological Processes, Molecular Functions, and Cellular Components in SRS

When a genome is sequenced or a protein is characterized, databases classify the information in different ways using automatic identification or manual annotation. In the UniProt database, an annotation is possibly made using the formal representation proposed in Gene Ontology (GO) under three aspects related to biological knowledge of the proteins: molecular function (MF), biological processes (BP), and cellular components (CC). In SRS, 3206 proteins were detected and identified, but 5962 MF, 2542 BP, and 1399 CC were obtained from their annotations in UniProt. Some proteins have more than one annotation and others have none [39].

**Figure 8:**
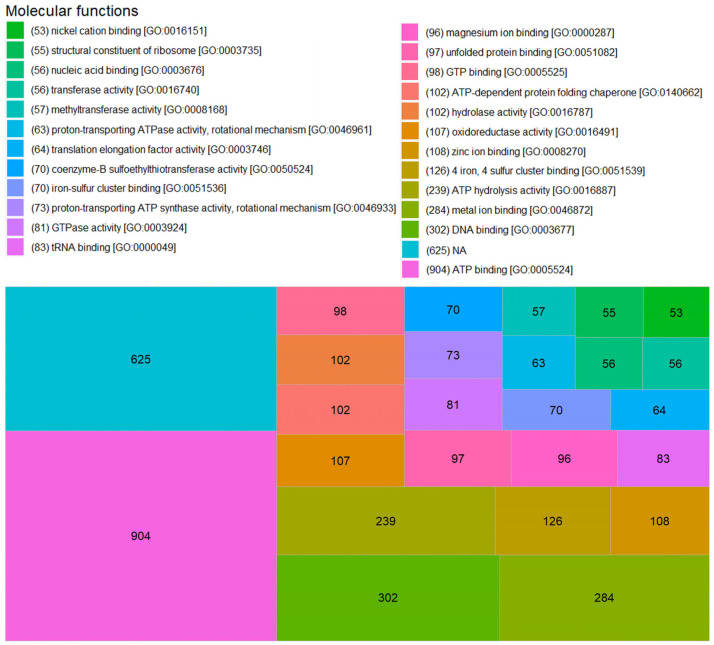
Molecular functions (MF) of annotated proteins detected in SRS. The first 25 MF are graphed. The GO identification corresponds to the Gene Ontology annotated for each protein in the UniProt database.

For MF, 478 different functions were obtained, but 625 proteins did not have any annotation (Figure 8). The most recurrent functions were ATP binding [GO:0005524] and DNA binding [GO:0003677] with 904 and 302 annotations, respectively. An example of an ATP-binding protein is the subunit α of V-type ATP synthase (A0A8G2FW98) of *Picrophilus oshimae* DSM 9789. As expected, this protein has other three annotations related to proton transport. Besides that, the DNA helicase (A0A811T524) of Ca. *Argoarchaeum ethanivorans* has the MF of DNA binding and ATP binding. Interestingly, metal ion binding [GO:0046872] had 284 annotations, and 126, 108, 96, 70, 53, and 45 annotations were obtained for the specific functions 4 iron, 4 sulfur cluster binding [GO:0051539], as well as zinc ion binding [GO:0008270], magnesium ion binding [GO:0000287], iron–sulfur cluster binding [GO:0051536], nickel cation binding [GO:0016151], and iron ion binding [GO:0005506], respectively. These MF are related to metalloproteins such as Mcr. Besides that, other relevant functions for methane production were coenzyme-B sulfoethylthiotransferase activity [GO:0050524] with 70 annotations, and methyltransferase activity [GO:0008168] with 57.

**Figure 9:**
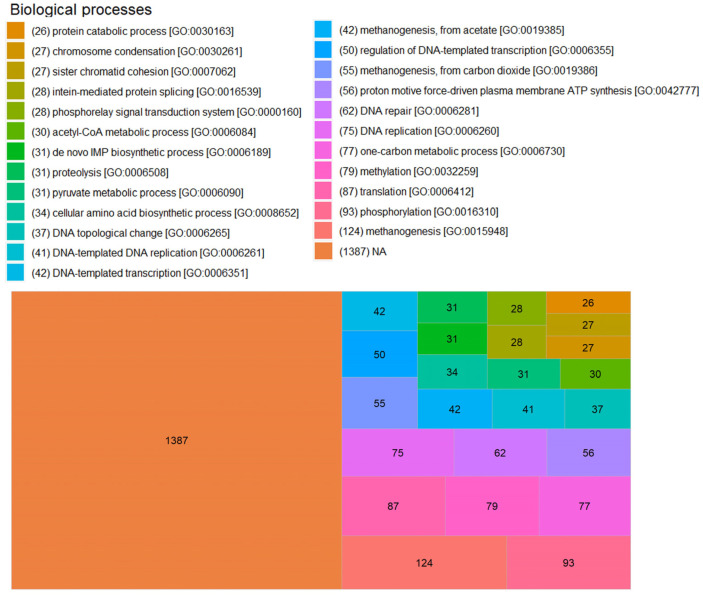
Biological processes of annotated proteins detected in SRS. The first 25 MF are graphed. The GO identification corresponds to the Gene Ontology annotated for each protein in the UniProt database.

For BP, 278 different processes were obtained, and the number of proteins without annotations increased to 1387 proteins. Methanogenesis [GO:0015948] was the most annotated process in SRS, with 124 annotations. An example for this BP is subunit B of formylmethanofuran dehydrogenase (A0A284VQ18) of Ca. *Methanoperedens nitroreducens*. This protein is related to the reversible reduction of ferredoxin, the formation of methanofuran, and the formation of CO_2_ from formylmethanofuran and water (reaction R03390 in KEGG), a grosso modo. This is related to methane production from CO_2_ (pathway module M00567 in KEGG). Some proteins have a specific BP annotation for methane production, such as methanogenesis from acetate [GO:0019385]. An example with this annotation is subunit δ of the acetyl-CoA decarbonylase/synthase complex (A0A4E0QXF6) of *Methanolobus halotolerans* (pathway module M00357 in KEGG). Other specific processes related to methane production were methylation [GO:0032259] with 79 annotations, one-carbon metabolic process [GO:0006730] with 77, methanogenesis from carbon dioxide [GO:0019386] with 55, and methanogenesis from acetate [GO:0019385] with 42 annotations (Figure 9).

**Figure 10:**
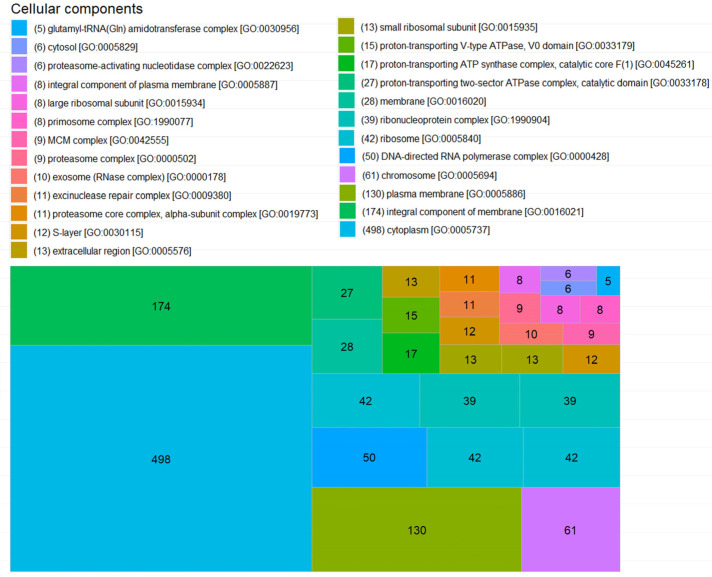
Cellular components of annotated proteins detected in SRS. The first 25 CC are graphed. Proteins with no annotation (NA) were removed to improve the presentation of this figure. The GO identification corresponds to the Gene Ontology annotated for each protein in the UniProt database.

For CC, 54 different component annotations were obtained, but 2107 proteins did have any annotation in this classification. Cytoplasm [GO:0005737] and cytosol [GO:0005829] components had 498 (Figure 10) and 6 annotations (Table S9), respectively. For membranes, more diverse annotations were obtained: an integral component of the membrane [GO:0016021] with 174, plasma membrane [GO:0005886] with 130, membrane [GO:0016020] with 28, and an intrinsic component of the plasma membrane [GO:0031226] with 1 (Table S9). An example of membrane CC annotation is the protein PAS domain S-box protein (A0A7J9SZJ7) of the ANME-2 archaeon cluster. This protein has two transmembrane helixes between amino acid residues 30–48 and 69–87 (features in UniProt).

**Figure 11:**
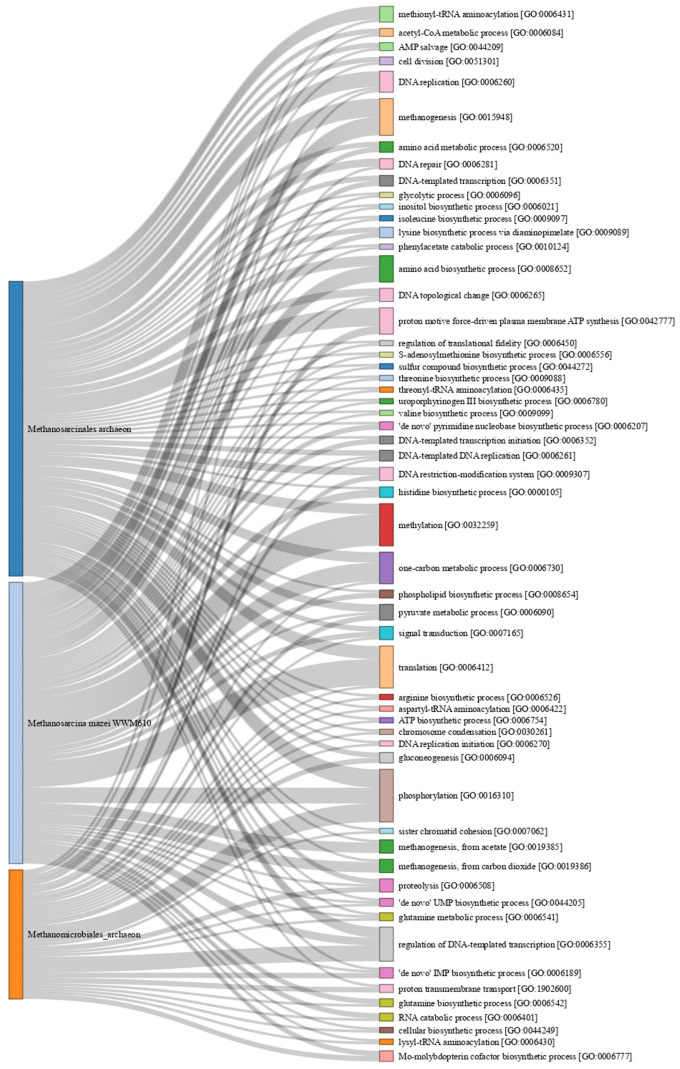
Biological process annotation for *Methanosarcinales archaeon*, *Methanosarcina mazei* WWM610, and *Methanomicrobiales archaeon*. Each branch corresponds to a specific protein and its annotation in the UniProt database (See Table S10). The Gene Ontology annotation is indicated in brackets. Only biological processes found in at least two proteins are shown.

On the other hand, we grouped the proteins of the three best-represented species *Methanosarcinales archaeon* (199 proteins), *Methanosarcina mazei* WWM610 (175 proteins), and *Methanomicrobiales archaeon* (80 proteins) to analyze the BP share of these organisms (Figure 11). We got 372 annotations from 252 proteins with BP annotations (Table S10). For *Methanosarcinales archaeon*, there were 101 proteins with 155 annotations, of which 112 had at least one shared BP with the other two organisms. Interestingly, we identified five proteins with the annotation acetyl-CoA biosynthetic process from acetate [GO:0019427], but it was not shared with either of the other two (Figure S2). For *M. mazei* WWM610, only 105 proteins were annotated; we identified 154 annotations, of which 47 were shared with the other two organisms. The most abundant proteins with no shared BP annotations were those associated with the carbohydrate metabolic process [GO:0005975], with four proteins (glycogen phosphorylase (A0A0E3LFC3), phosphomannomutase (A0A0E3LG81), glucoamylase (A0A0E3PVI0), and a probable phosphoglucosamine mutase (A0A0E3PX89); Figure S2). Finally, for *Methanomicrobiales archaeon,* we identified 46 annotated proteins with 63 annotations and 14 proteins with no shared annotations. The most abundant proteins with no shared BP annotations were those related to the conserved phosphorelay signal transduction system [GO:0000160].

Besides that, the most abundant BP annotation was phosphorylation [GO:0016310] with 20 proteins. The PAS domain S-box sensory proteins of *Methanomicrobiales archaeon* were the most abundant for this annotation (A0A7K3Z0P0, A0A7K3YGI8, A0A7L4PYV9, and A0A7K4CA95). For methanogenesis [GO:0015948], 14 proteins with this BP annotation were identified; for Mcr, only subunit γ of *Methanosarcinales archaeon* (A0A7J4EMK2) and subunit β of *M. mazei* WWM610 (A0A0E3PU26) were observed. Interestingly, we identified five proteins for methanogenesis from carbon dioxide [GO:0019386] and five for methanogenesis from acetate [GO:0019385], but no annotation of this kind for *Methanomicrobiales archaeon*. For methylation [GO:0032259], 16 proteins with this BP annotation were identified. The previously mentioned MtaB was the type of protein most abundant in this BP annotation (A0A0E3PSS7, A0A0E3PYZ2, A0A0E3LEL4, A0A0E3LF38, and A0A0E3LFN7). For the one-carbon metabolic process [GO:0006730], 12 proteins were identified as being subunits H, C, and G of tetrahydromethanopterin S-methyltransferase (A0A0E3Q199, A0A0E3LG77, and A0A7K4B3Y9) of *M. mazei* WWM610. For *Methanomicrobiales archaeon*, only adenosyl homocysteinase (A0A7K4B3Y9) was detected with a BP annotation.

## 4. Discussion

The preferred composition of biogas is a high content of hydrogen or methane, perhaps both. This ensures rapid oxidation of the biogas, releasing high heat energy that can be coupled to various anthropic processes. The establishment of control states that favor biological growth in bioreactors is on its way to a steady state. For our initial growth conditions, this state for biogas production is reached and changed in a reconciling mathematical curve (Figure 2). Although the pattern found in our best-represented proteins suggests that the metabolism of methanol, formate, and acetyl-CoA are established and phosphorylation by V-ATP synthase is also present (Figure 3), the low biogas quality showed that methane metabolism is not privileged in SRS. The above could be attributed to a different condition in this first experiment: the consortia in SRS are not accustomed to these initial experimental conditions [66]. The change from an oligotrophic site in nature to a controlled system with a complete growth medium, including thermal and osmotic shock, substrate/cofactor/inoculum ratios, and mechanical agitation to ensure homogeneity [42], as well as a sufficient abundance of organisms that preferentially produce hydrogen or methane, but not H_2_S and CO_2_ [67], among others. In the case of the present study, perhaps in addition, the adaptability of the archaeal consortia in high-arsenic sediments and methanotrophic species could be of importance.

Interestingly, the archaeal machinery of arsenic metabolism has not been detected [68]. The water quality of the Santiago River is monitored by the State Water Commission of the State of Jalisco México [69]. They report 52 physicochemical and biological parameters, including heavy metals. This information is contained in a database that begins in 2003 and continues to the present. During this period, five water bodies in Jalisco were monitored monthly at different sites, including the Rio Santiago. For the sediment-sampling site (Figure 1), a maximum of 19.9 μg/L arsenic was recorded on July 1, 2021. This suggests that although the inorganic fraction in the sediment contains a significant concentration of arsenic, the water found in the interface contains a relatively high concentration [70]. This environment does not promote an expression of protective metabolism for arsenic in SRS and could not be the reason for the low-quality biogas produced [71], but some proteins, such as thermosome subunits and DnaK proteins (Figure 5), have been found in similar conditions [72].

In SRS, methanotrophic archaea represented only 5.6% of all species found, and their proteins represented 7.7% of the total proteins (Figure 5). However, only three methanotrophic Mcr proteins were detected, suggesting that methane oxidation is not an abundant biological process in SRS and its contribution to low biogas quality is minimal. Interestingly, the three species with the most detected proteins (Figure 11) have a similar number of proteins related to phosphorylation, but only the first and second have the same number of proteins related to methanogenesis, and the third has only one protein related to the one-carbon metabolic process. This suggests that under our conditions not all species are significant methanogens. For the third, the most identified BP was the regulation of DNA template transcription.

Factors such as completeness of annotation and amount of information affect the bioinformatic analysis of proteomes. In this work, 464 proteins without annotation were found. This represents 14.4% of the total detected proteins. This type of work requires greater integration of the data and graphical forms of representation, although the molecular process is known. The biological function to which it belongs, and its cellular location allows us to have a global view of the metabolic pathways established in the growth of SRS, it is necessary to have the support of a curated database to reduce the bias of the information. It could be assumed as obvious that the dominant MF in archaeal cells is related to the bioenergetics of ATP, in this study we found that ATP binding is the most detected MF, three times more than the second function, DNA binding (Figure 8). For BP, as expected, methanogenesis is the most annotated, with some authors even describing exactly what type of methanogenesis it is (methanol, acetate, etc.). However, the lack of information starts to become relevant as only 57% of the detected proteins have an annotation (Figure 9). In SRS, the cellular distribution of the detected proteins is balanced between cytoplasm and membrane, as in the previous case, the lack of annotations is relevant with more than 63% of the proteins without annotations (Figure 10). Further proteomic studies are needed to analyze the results obtained so far and to make comparisons of the dominant state of the functions, processes and components involved in the homeostasis of microbial consortia.

## 5. Conclusions

The low methane concentration in the biogas of the first experiment indicated that the performance of SRS as an inoculum to produce methane is not good enough. However, the subsequent proteomic analysis suggests that in SRS, there are diverse euryarcheotic species with a high potential for methane production. The abundance of members of the order *Methanosarcinales* could be important to improve methane production; enrichment of these species in the inoculum using culture medium directed to favor the growth of these organisms could be a first approach.

On the other hand, some proteins were identified by a relatively large number of peptides, although their abundance in terms of mass was not the greatest. This suggests that peptide digestion is efficient for these proteins, which could be used as an identification marker for these species (i.e., MtaB to identify *Methanosarcina mazei* WWM610). Besides that, the abundance of certain proteins in SRS suggests that some metabolisms are established and correspond to nutrients in the culture medium and growth conditions. The stress imposed by the change in the environment of the SRS and the relative abundance of carbon sources promote its use with the consequent processing of ATP and DNA.

In SRS, some species are relevant not only because of their capacity to produce methane. For example, the genus *Methanobrevibacter* and its abundant adhesin-like proteins could be advantageous for the formation of a physical structure for consortia, and syntrophic relations between not only archaea, but also bacteria. In addition, in bioreactors it is desirable to have granular consortia; this facilitates the treatment of wastewater and the subsequent separation of treated water and granular sludge.

The diversity of metabolisms found in SRS gave us insight into the complex interactions between archaeal species. Something relevant was the presence of methanotrophic archaea. Perhaps their presence in bioreactors for biogas production is not desirable; however, in nature, they can alleviate part of the problem of uncontrolled methane generation. These uncultured methanotrophs and the presence of little more than 8% of expressed uncharacterized proteins in SRS show a potential research area. It is necessary to say that the Santiago River is a natural resource that requires an urgent intervention to recover its natural conditions to support a healthy ecosystem.

## Figures and Tables

**Table 1:** Physicochemical characterization of sediments from the Santiago River.

Parameter	Units	Value
Temperature ^1^	°C	16
pH ^1^	-	6.88
Electric conductivity ^1^	μS/cm	8305
ORP ^1^	mV	−190
Dissolved oxygen	mg/L	0.9
TDS ^1^	g/L	4.87
Salinity ^1^	ppt	4.1
Total nitrogen	mg/L	800 ± 16
Total COD	mg/L	34,200 ± 46
Soluble COD	mg/L	237 ± 5
TOC	mg/L	800 ± 21
TS	g/L	76.83 ± 0.08
VS	g/L	58.88 ± 0.1
Cd	mg/kg	0.198 ± 0.05
Co	mg/kg	12.033 ± 1.49
Fe	mg/kg	19,761.7 ± 1839.1
Mg	mg/kg	0.03 ± 0.006
Cr	mg/kg	104.68 ± 8.13
As	mg/kg	960 ± 55.717

^1^ Determined in situ.

## Data Availability

All the data are reported in the manuscript.

## Supplementary Materials

The following supporting information can be downloaded at: https://www.mdpi.com/article/10.3390/microorganisms11071640/s1, Figure S1: SDS-PAGE of whole cell extract; Figure S2. Sankey diagram of all biological process annotation detected from UniProt of *Methanosarcinales archaeon*, *Methanosarcina mazei* WWM610, and *Methanomicrobiales archaeon*; Table S1. The first 100 more abundant proteins in SRS; Table S2. The first 100 best represented proteins in SRS; Table S3. V-type ATP synthase subunits detected in SRS; Table S4. Orthologs identified in SRS; Table S5. Acetyl-CoA decarbonylase subunits detected in SRS; Table S6. Methyl-coenzyme M subunits detected in SRS; Table S7. Proteins related with biosynthetic pathways of coenzyme B and M; Table S8. Proteins detected of ANME species; Table S9. Total annotations of functions from UniProt; Table S10. Total biological process annotation from UniProt of *Methanosarcinales archaeon*, *Methanosarcina mazei* WWM610, and *Methanomicrobiales archaeon*.
