# Supplementary figures and images for: An Initial Proteomic Analysis of Biogas-Related Metabolism of Euryarchaeota Consortia in Sediments from the Santiago River, México
Source: Microorganisms. 2023 Jun 23;11(7):1640. doi: 10.3390/microorganisms11071640 (PMC10384328; doi:10.3390/microorganisms11071640)

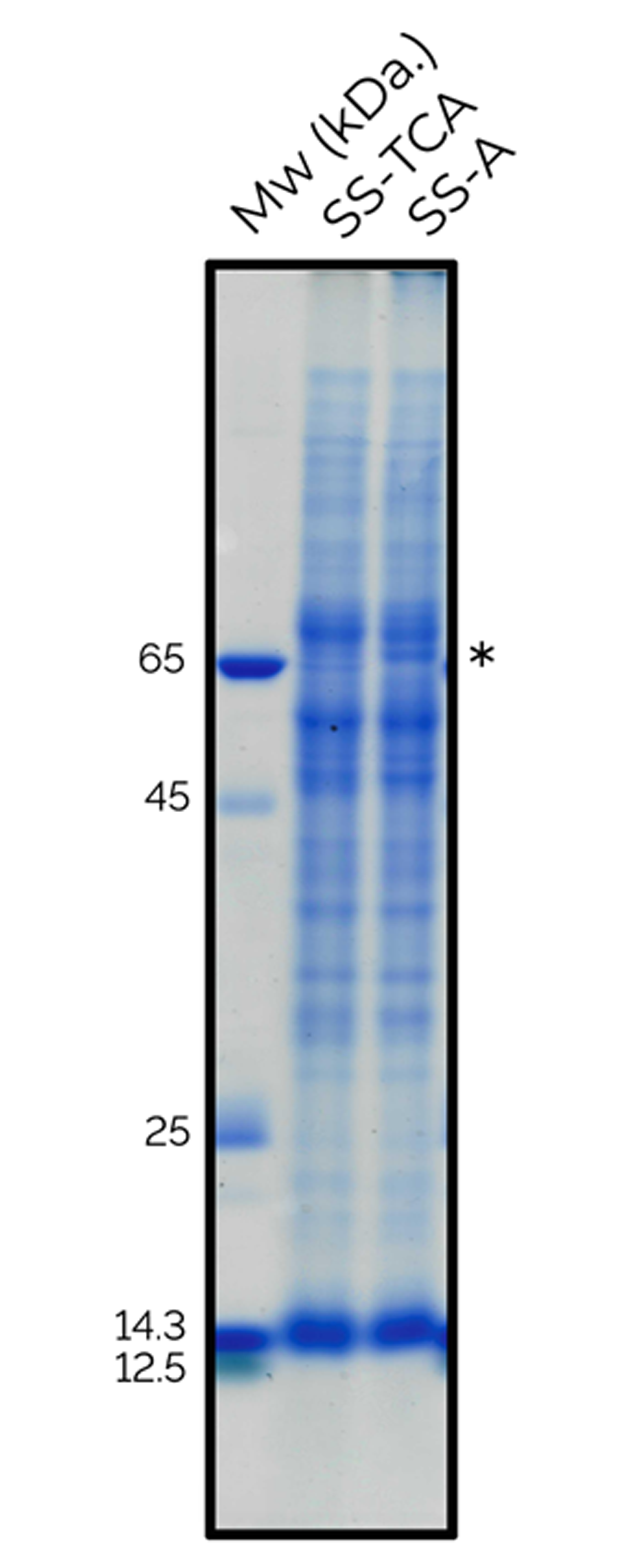

Supplement: Supplementary file 1 [file microorganisms-11-01640-s001.zip › Figure S2.tif]

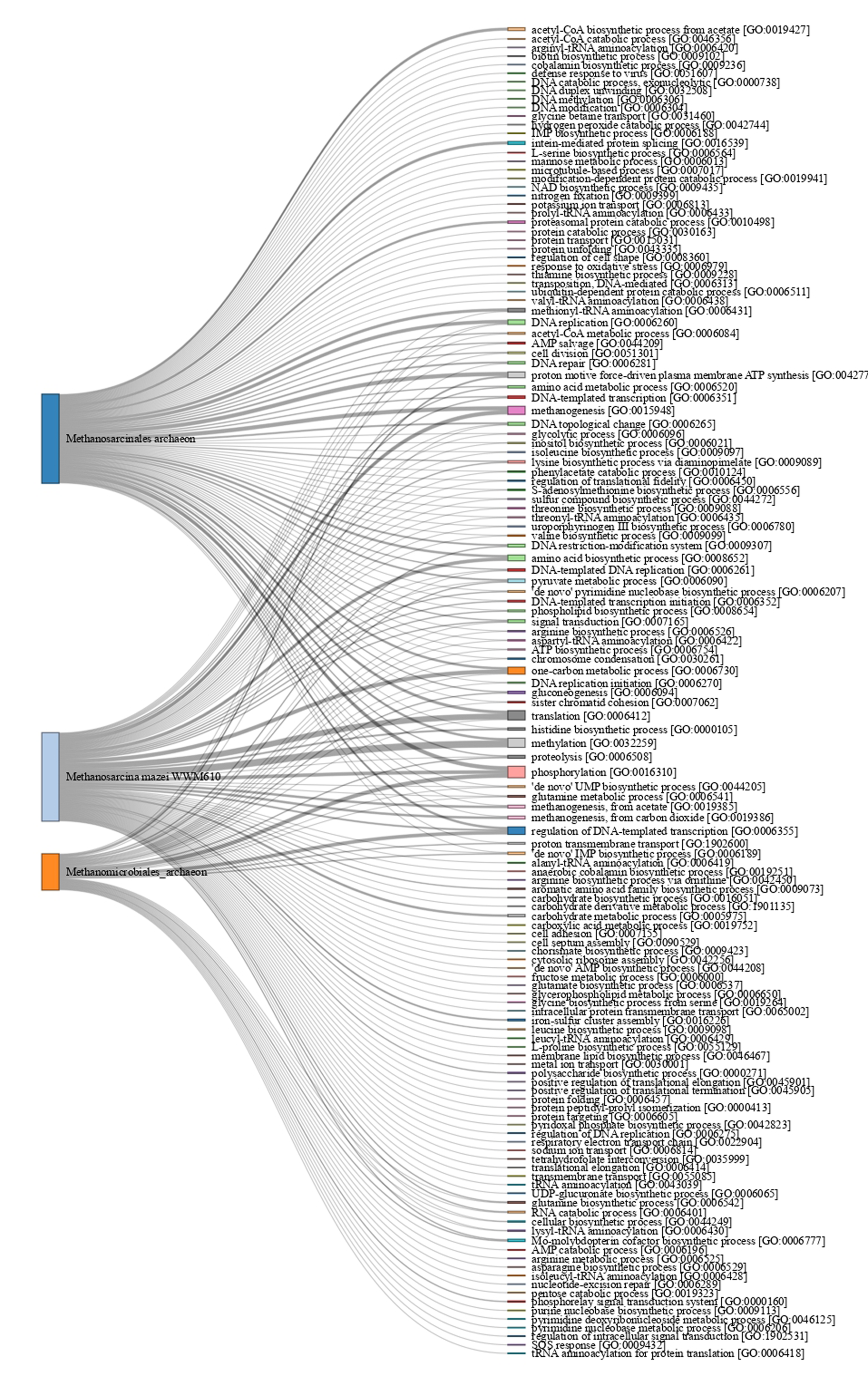

Supplement: Supplementary file 1 [file microorganisms-11-01640-s001.zip › Fig. S3.tif]
